# Supplementary material for: Selective Inhibition of Genomic and Non-Genomic Effects of Thyroid Hormone Regulates Muscle Cell Differentiation and Metabolic Behavior
Source: Int J Mol Sci. 2021 Jul 2;22(13):7175. doi: 10.3390/ijms22137175 (PMC8269436; doi:10.3390/ijms22137175)
Supplement: Supplementary file 1 [file ijms-22-07175-s001.zip › ijms-1229699-supplementary.pdf]

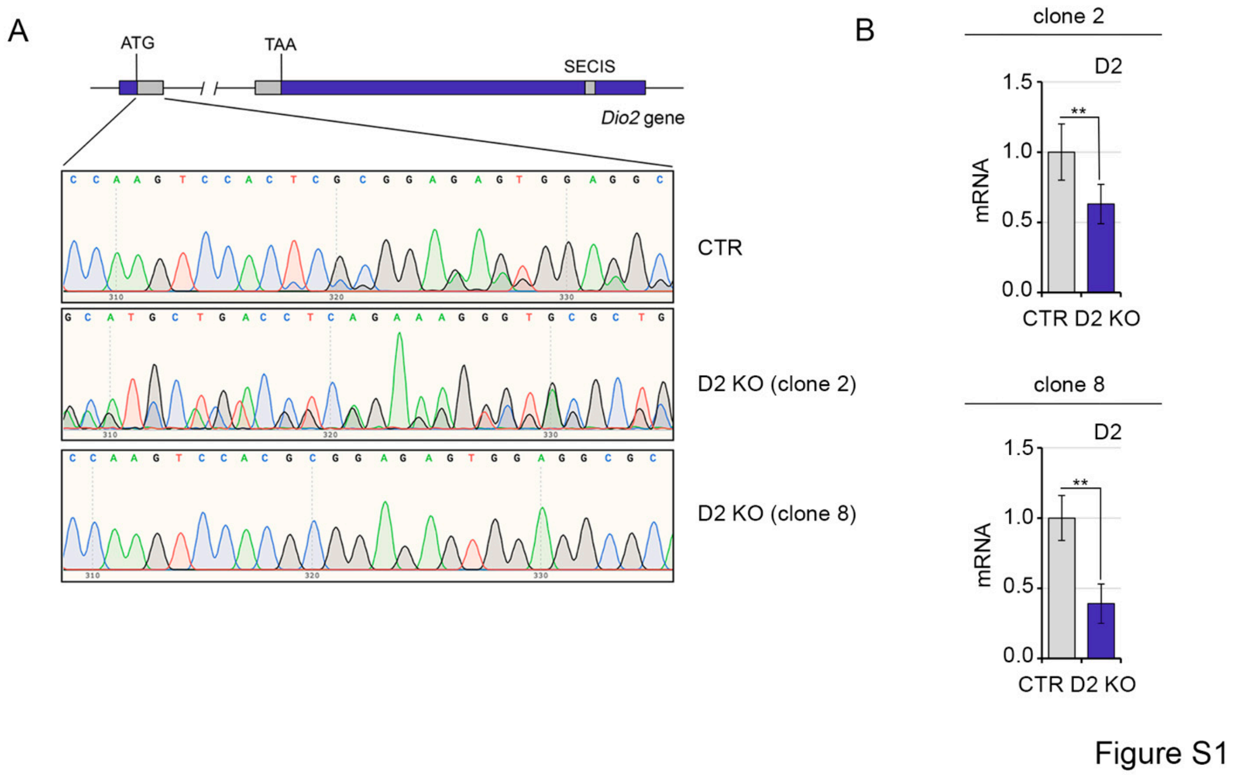

Figure S1. Mutagenesis of *Dio2* gene in C2C12 cells. (**A**) Schematic representation of *Dio2* locus. Mutagenesis of *Dio2* locus was assessed by genomic DNA sequencing of exon 1. Electropherogram of two different D2 KO clones (clone 2 and clone 8). (**B**) D2 expression was evaluated by Real-Time PCR in D2 KO (clone 2 and clone 8) and CTR cells. $^{**}p < 0.01$.

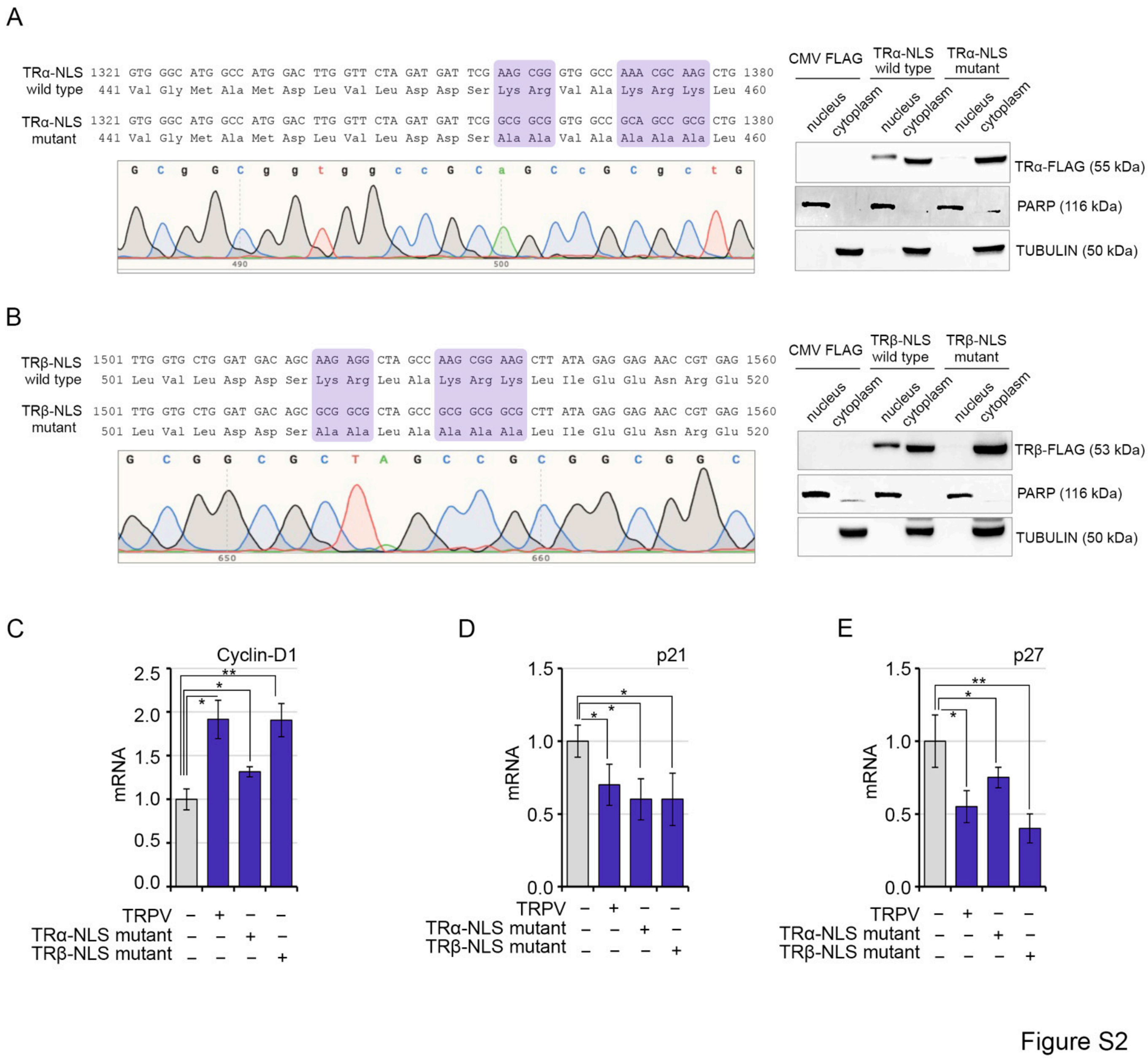

Figure S2. Block of nuclear translocation of TRs enhances cell proliferation. (**A**) Comparison of FLAG-TRα-D wt and FLAG-TRα-D mut sequences. The expression vector CMV FLAG, FLAG-TRα-D wt and FLAG-TRα-D mut plasmids were transfected into C2C12 cells as described in Materials and Methods. Nuclear and cytoplasmic localization of TRα-FLAG was evaluated by Western Blot analysis of nuclear and cytoplasmic fraction of cell lysate. PARP expression was measured as nuclear loading control, while Tubulin expression was measured as cytoplasmatic loading control. (**B**) Comparison of schematic FLAG-TRβ-D wt and FLAG-TRβ-D mut sequences.

The expression vector CMV FLAG, the FLAG-TRβ-D wt and FLAG-TRβ-D mut plasmids were transfected into C2C12 cells as described in Materials and Methods. Nuclear and cytoplasmic localization of TRβ-FLAG was evaluated by Western Blot analysis of nuclear and cytoplasmic fraction of cell lysate. PARP expression was measured as nuclear loading control, while Tubulin expression was measured as cytoplasmatic loading control. **(C-E)** Cyclin-D1, p21 and p27 expression was evaluated by Real-Time PCR in C2C12 cells transiently transfected with a TRPV, FLAG-TRα D mut and FLAG-TRβ-D mut expressing vector or the CMV FLAG plasmid (control). Data represent the mean of three independent experiments performed in duplicate. *$p < 0.05$, **$p < 0.01$.

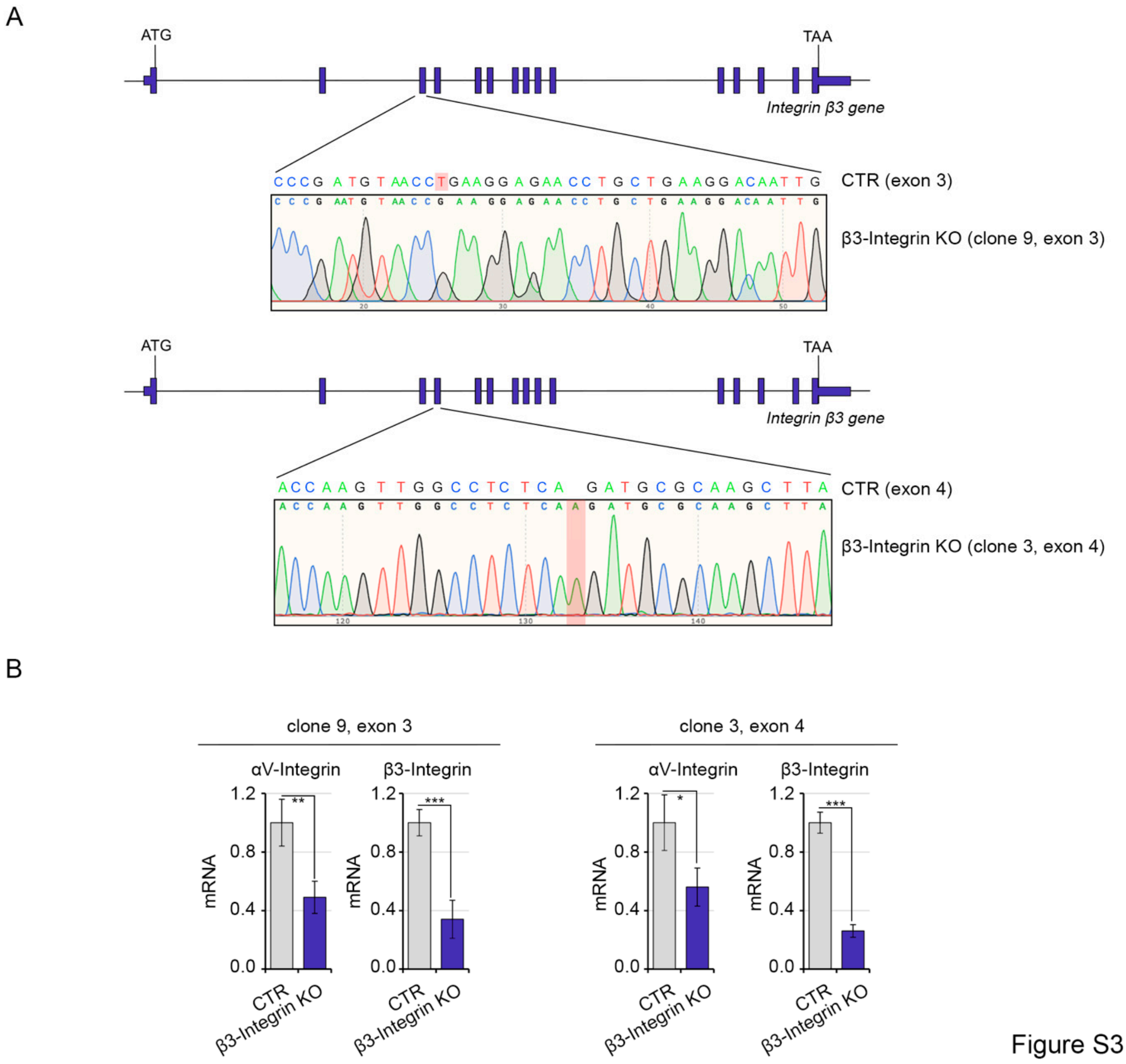

Figure S3

Figure S3. Mutagenesis of β3-Integrin (ITGβ3) gene in C2C12 cells. (**A**) Schematic representation of β3-Integrin (ITGβ3) locus. Mutagenesis of β*3-Integrin* (*ITG*β*3*) locus was assessed by genomic DNA sequencing of exon 3 (clone 9) and exon 4 (clone 3). Electropherogram of two different β3-Integrin KO clones (clone 9, exon 3 and clone 3, exon 4). (**B**) αV-Integrin and β3-Integrin expression was evaluated by Real-Time PCR in β3-Integrin KO (clone 9, exon 3 and clone 3, exon 4) and CTR cells.

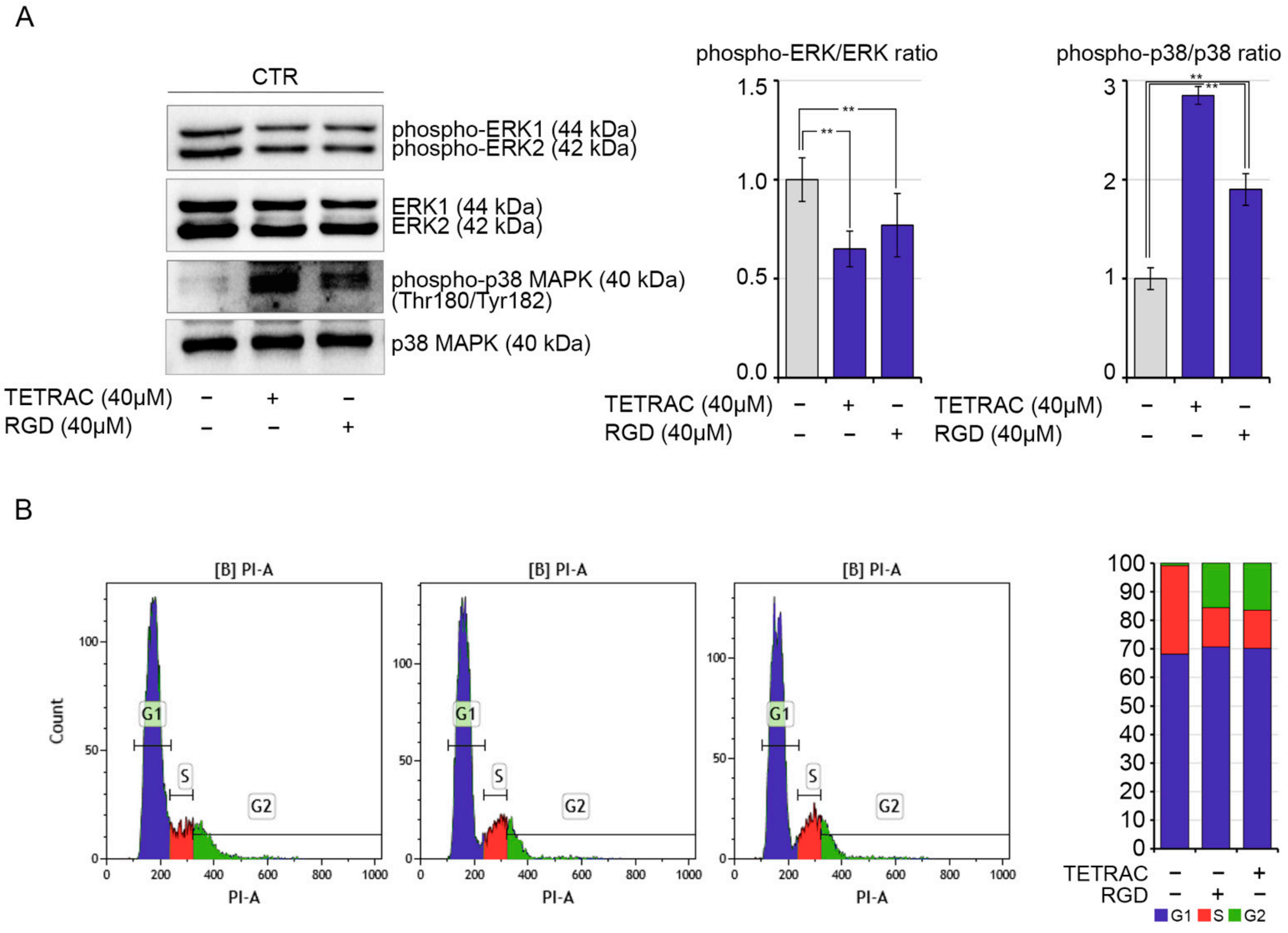

Figure S4

Figure S4. Drug-mediated reduction of Integrin signaling reduces cell proliferation (**A**) Western Blot analysis of phospho-ERK 1/2 and phospho-p38 MAPK in CTR cells treated with 40.0 μM TETRAC or 40.0 μM RGD. ERK 1/2 and p38 MAPK expression was measured as a loading

control. Quantification of the protein levels of phospho-ERK 1/2 versus ERK 1/2 and phospho-p38 MAPK versus p38 MAPK levels are represented by histograms. Data represent the mean of three independent experiments. (**B**) Cell cycle distribution was measured in CTR cells at 6 hours after treatment with 40.0 µM TETRAC or 40.0 µM RGD. Cells were analyzed with flow cytometry after propidium iodide staining (n=3).

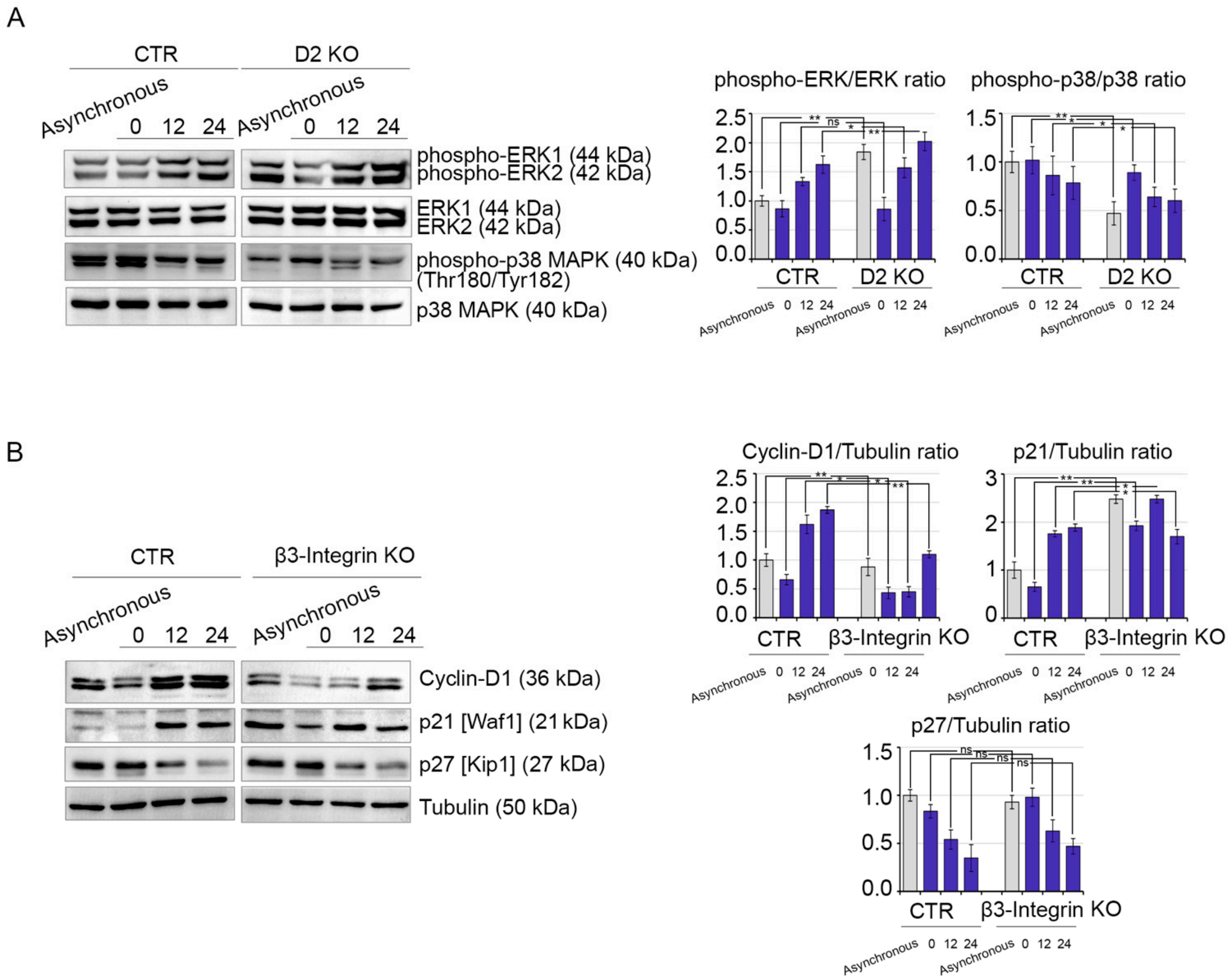

Figure S5

Figure S5. Impact of D2 depletion on ERK 1/2 and p38 MAPK signaling and the influence of β3-Integrin knock-out on Cyclin-D1, p21 and p27 signaling. (**A**) Western Blot analysis of phospho-ERK 1/2 and phospho-p38 MAPK in asynchronized and synchronized D2 KO and CTR cells after cell cycle re-initiation at 0, 6, 12 and 24 hours after release from serum starvation. ERK 1/2 and p38

MAPK expression was measured as a loading control. Quantification of the protein levels of phospho-ERK 1/2 versus ERK 1/2 and phospho-p38 MAPK versus p38 MAPK levels are represented by histograms. Data represent the mean of three independent experiments. (**B**) Western Blot analysis of Cyclin-D1, p21 and p27 in asynchronized and synchronized β3-Integrin KO and CTR cells after cell cycle re-initiation at 0, 6, 12 and 24 hours after release from serum starvation. Tubulin expression was measured as a loading control. Quantification of the protein levels of Cyclin-D1, p21 and p27 versus Tubulin levels is represented by histograms. Data represent the mean of three independent experiments. *$p < 0.05$, **$p < 0.01$.

Table 1: List of antibodies

Antibodies used for Western Blot and Immunofluorescence analysis			
**Antibody**	**Source**	**Identifier**	**Diluition**
α-Tubulin	Sigma Aldrich	T8203	1:5000 WB
α-FLAG M2	Sigma Aldrich	F3165	1:1000 WB
Cyclin-D1 (HD11)	Santa Cruz	sc-246	1:1000 WB
Myosin heavy chain (MHC)	Hybridoma Bank	MF20	1:50 IF
MyoD (C-20)	Santa Cruz	sc-304	1:1000 WB
Myogenin (M-225)	Santa Cruz	sc-576	1:1000 WB
ERK1/2 (K-23)	Santa Cruz	sc-94	1:1000 WB
phospho-p44/42 MAPK (Erk1/2) (Thr202/Tyr204)	Cell Signaling Technology	#4370	1:5000 WB
PARP	Cell Signaling Technology	#9542S	1:1000 WB
p38 MAPK	Cell Signaling Technology	#9211	1:1000 WB
phospho-p38 MAPK (Thr180/Tyr182)	Cell Signaling Technology	#9211	1:1000 WB
p21 (C-19)	Santa Cruz	sc-397	1:1000 WB
p27 Kip1	BD Biosciences	610242	1:1000 WB

Table 2: List of oligonucleotides

**Oligonucleotides used for Real-Time PCR**		
**Gene Symbol**	**Oligos**	**Sequence**
Ccnd1	mCyclin-D1_F	GCTCCTGTGCTGCGAAGTGGA
	mCyclin-D1_R	TCATGGCCAGCGGGAAGACCT
Cdkn1a	mp21_F	ACAAGAGGCCCAGTACTTCC
	mp21_R	GGGCACTTCAGGGTTTTCTC
Cdkn1b	mp27_F	GCGGTGCCTTTAATTGGGTC
	mp27_R	GTTGGCCCTTTTGTTTTGCG
Dio2	mDio2_F	CTTCCTCCTAGATGCCTACAAAC
	mDio2_R	GGCATAATTGTTACCTGATTCAGG
Gclc	mGCLC_F	CCTGGAGCCTCTGAAGAACA
	mGCLC_R	AGACTCGTTGGCATCATCCA
Gpx1	mGPX_F	CTCGGTTTCCCGTGCAATCAG
	mGPX_R	GTGCAGCCAGTAATCACCAAG
Hmox1	mHO1_F	TTCAGAAGGGTCAGGTGTCC
	mHO1_R	CAGTGAGGCCCATACCAGAA
ItgaV	maV-Integrin_F	GATTCGCCGTGGACTTCTTC
	maV-Integrin_R	ATCAAACTCAATGGGCTGGC
Itgb3	mb3-Integrin_F	GATTACCGACCCTCTCAGCA
	mb3-Integrin_R	GTCCCCACAGTTACATTGCC
Myh7	mMHC-I_F	CGCTCCACGCACCCTCACTT
	mMHC-I_R	GTCCATCACCCCTGGAGAC
Myh4	mMHC-IIb_F	GCTAGGGTGAGGGAGCTTGAA
	mMHC-IIb_R	AGACCCTTGACGGCTTCGA
Myod1	mMyod_F	GACCTGCGCTTTTTTGAGGACC
	mMyoD_R	CAGGCCCACAGCAAGCAGCGAC
Myog	mMyogenin_F	TTGCTCAGCTCCCTCAACCAGGA
	mMyogenin_R	TGCAGATTGTGGGCGTCTGTAGG
Nfe2l2	mNFE2L2_F	TGCCCACATTCCCAAACAAG
	mNFE2L2_R	CTGCCAAACTTGCTCCATGT
Ppia	mCyA_F	CGCCACTGTCGCTTTTCG
	mCyA_R	AACTTTGTCTGCAAACAGCTC
Sod2	mSOD2_F	ATCAGGACCCATTGCAAGGA
	mSOD2_R	AGGTTTCACTTCTTGCAAGCT

**Oligonucleotides used for mutagenesis**	
mTRα-MUT_F	CTAGATGATTCGGCGGCGGTGGCCGCAGCCGCGCTGATTGAGCAG
mTRα-MUT_R	CTGCTCAATCAGCGCGGCTGCGGCCACCGCCGCCGAATCATCTAG
mTRβ-MUT_F	CTGGATGACAGCGCGGCGCTAGCCGCGGCGGCGCTTATAGAGG
mTRβ-MUT_R	CCTCTATAAGCGCCGCCGCGGCTAGCGCCGCGCTGTCATCCAG

Figure 1E

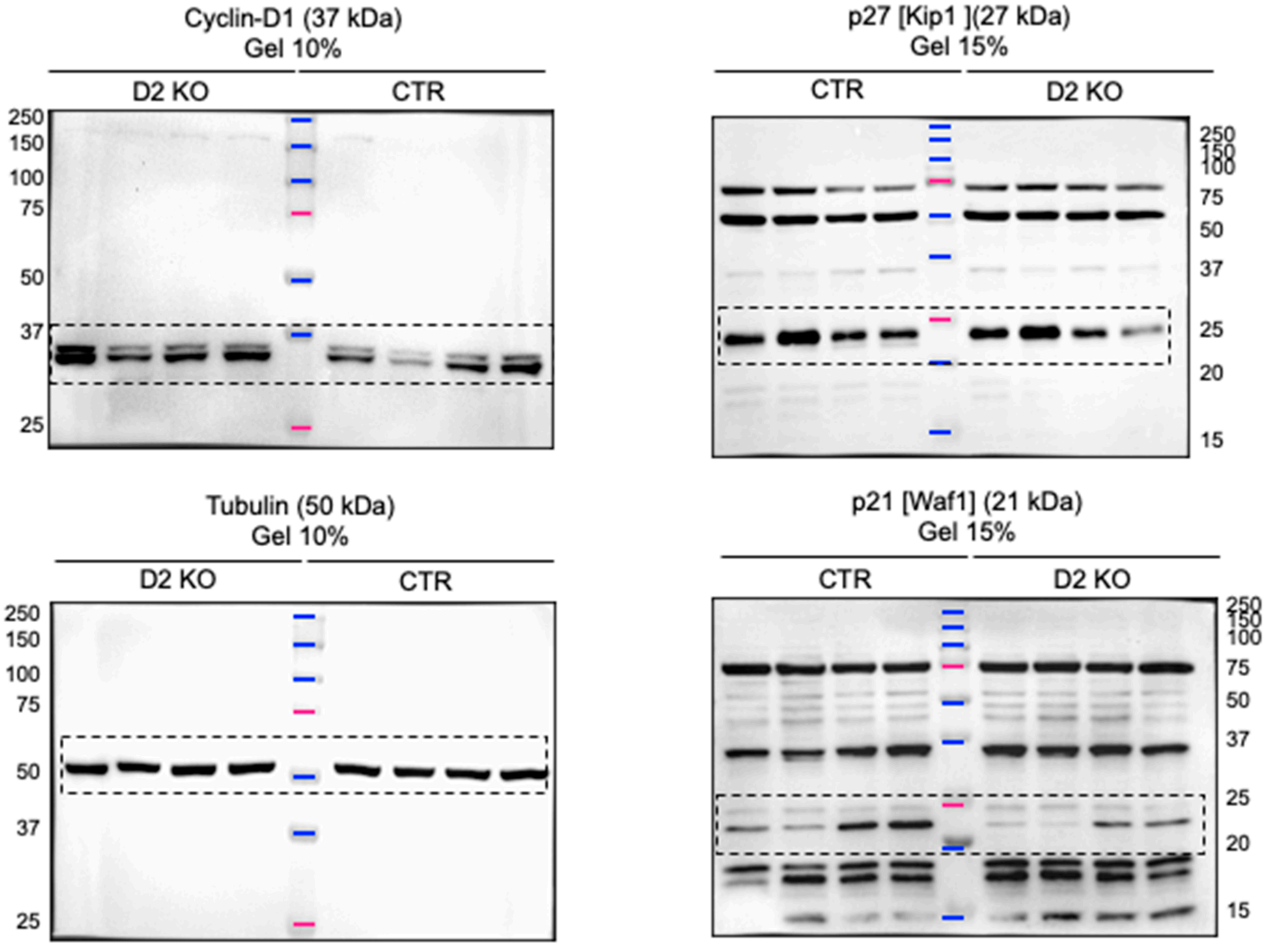

Precision Plus Protein Standards:
Dual Color BIORAD #161-0374

Figure 2D

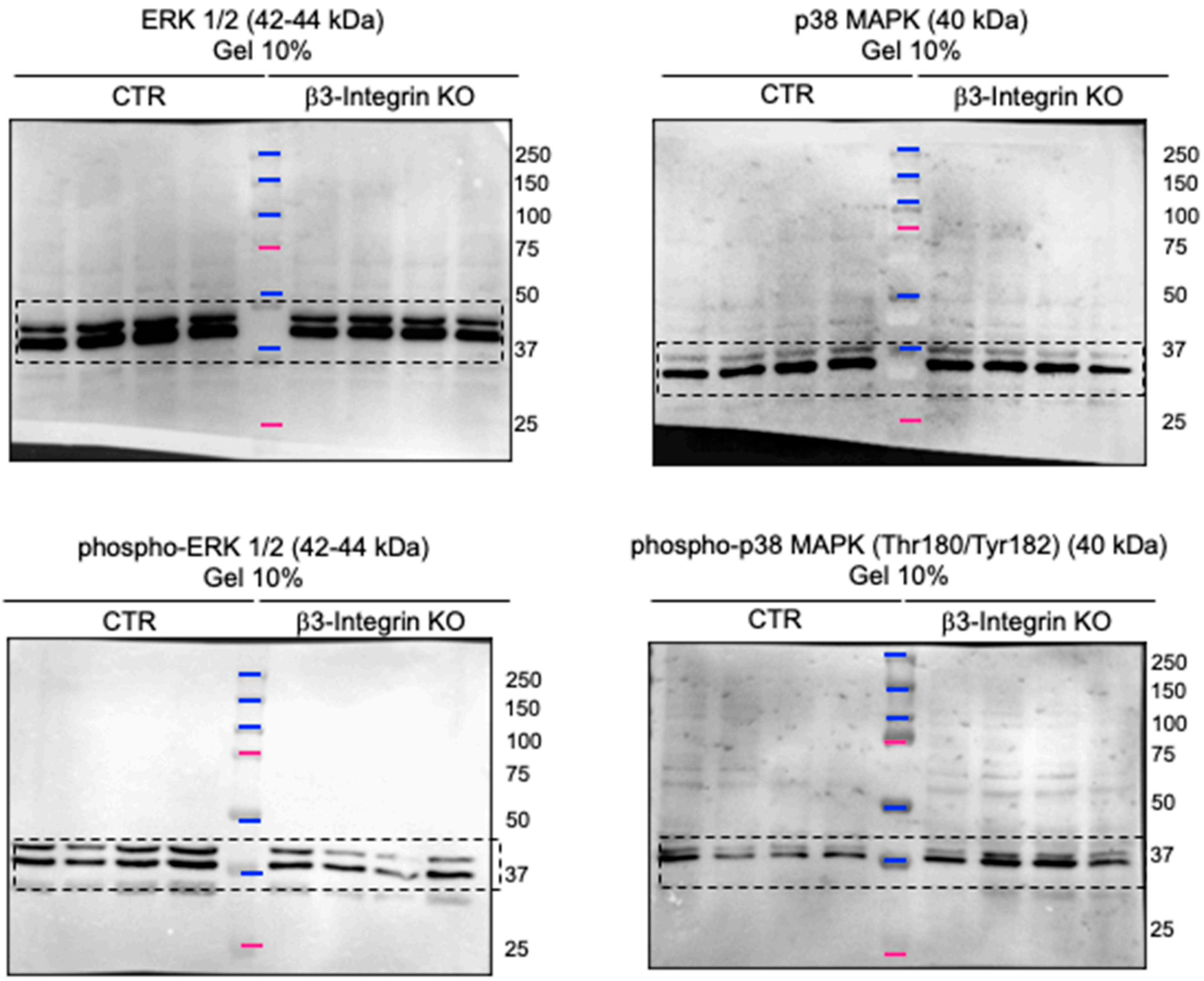

Precision Plus Protein Standards:
Dual Color BIORAD #161-0374

Figure 3C

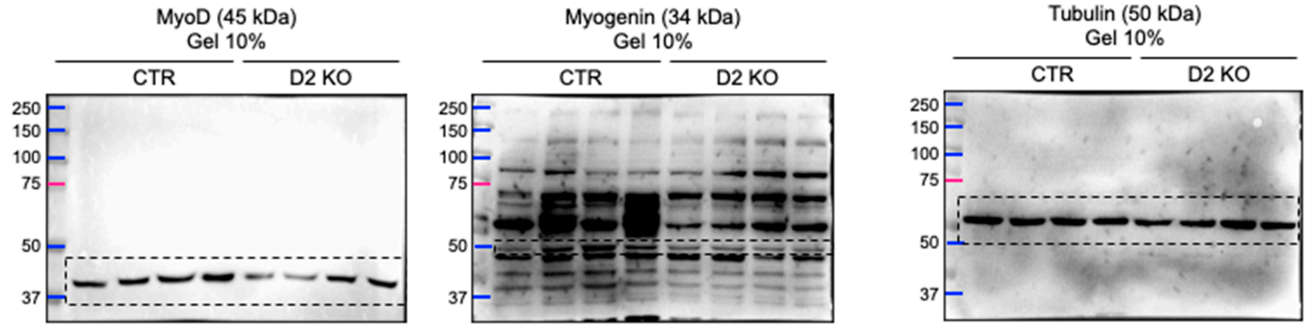

Figure 3I

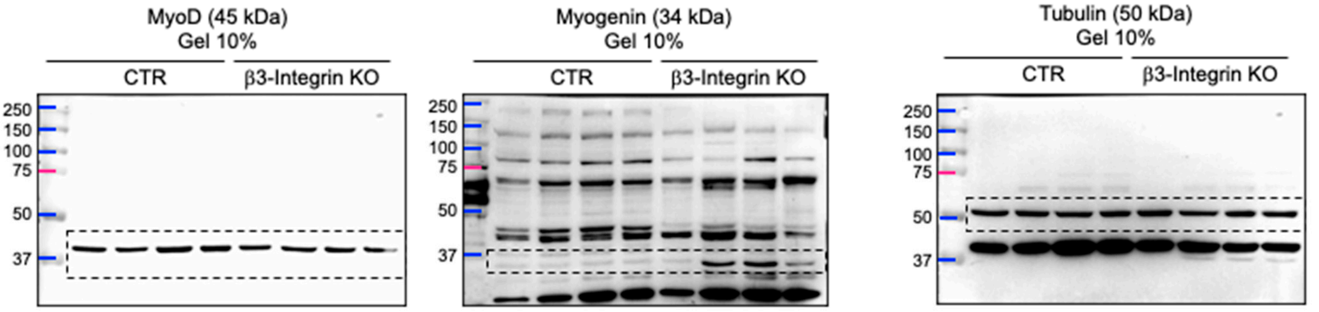

Precision Plus Protein Standards:
Dual Color BIORAD #161-0374

Figure S2A

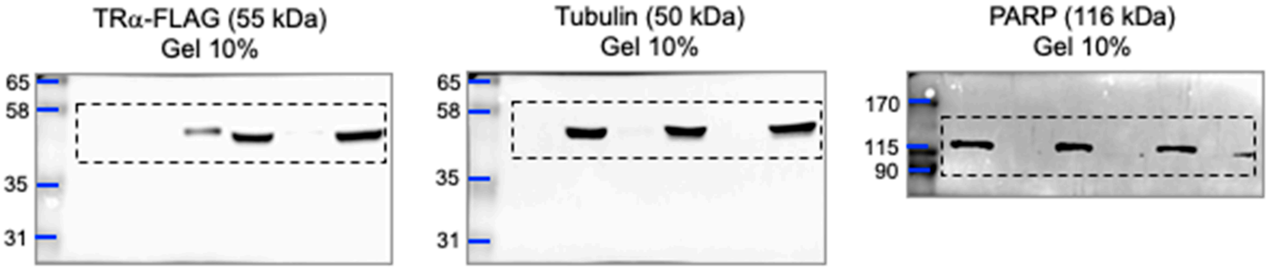

Figure S2B

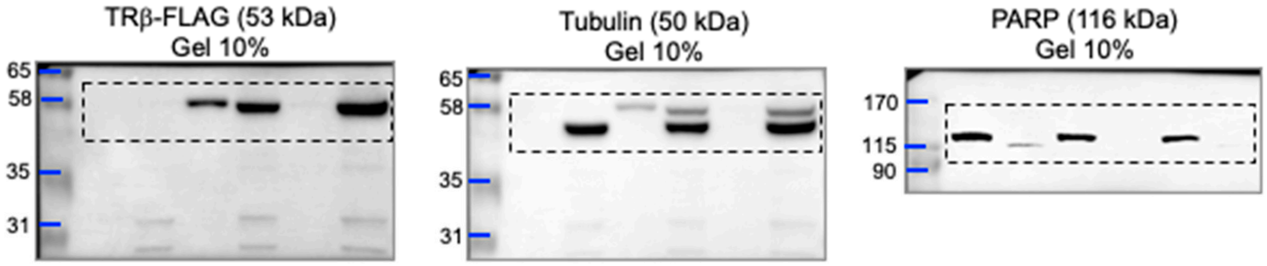

Marker Sigma
cod. SDS7B2

Figure S4A

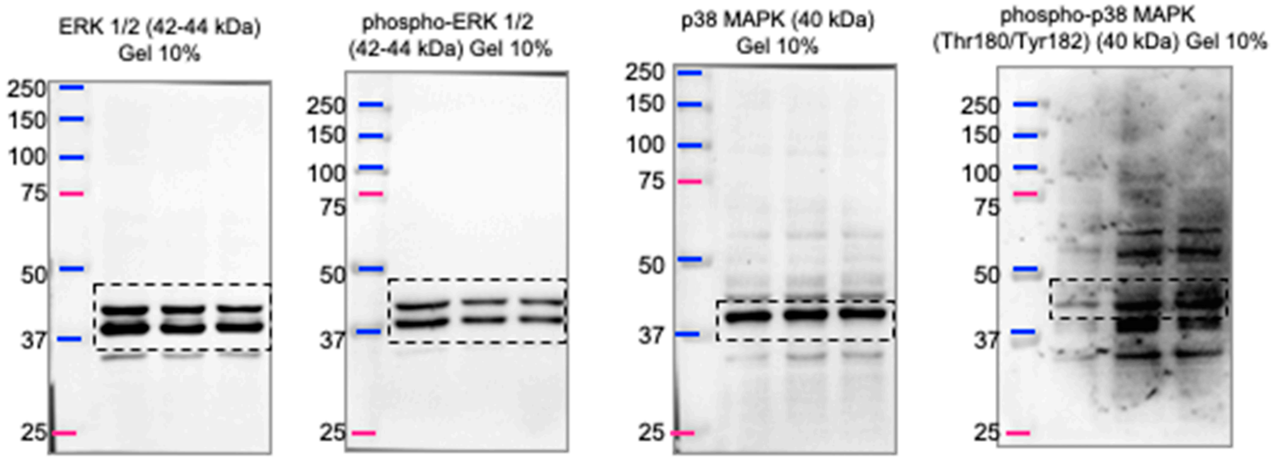

Precision Plus Protein Standards:
Dual Color BIORAD #161-0374

Figure S5A

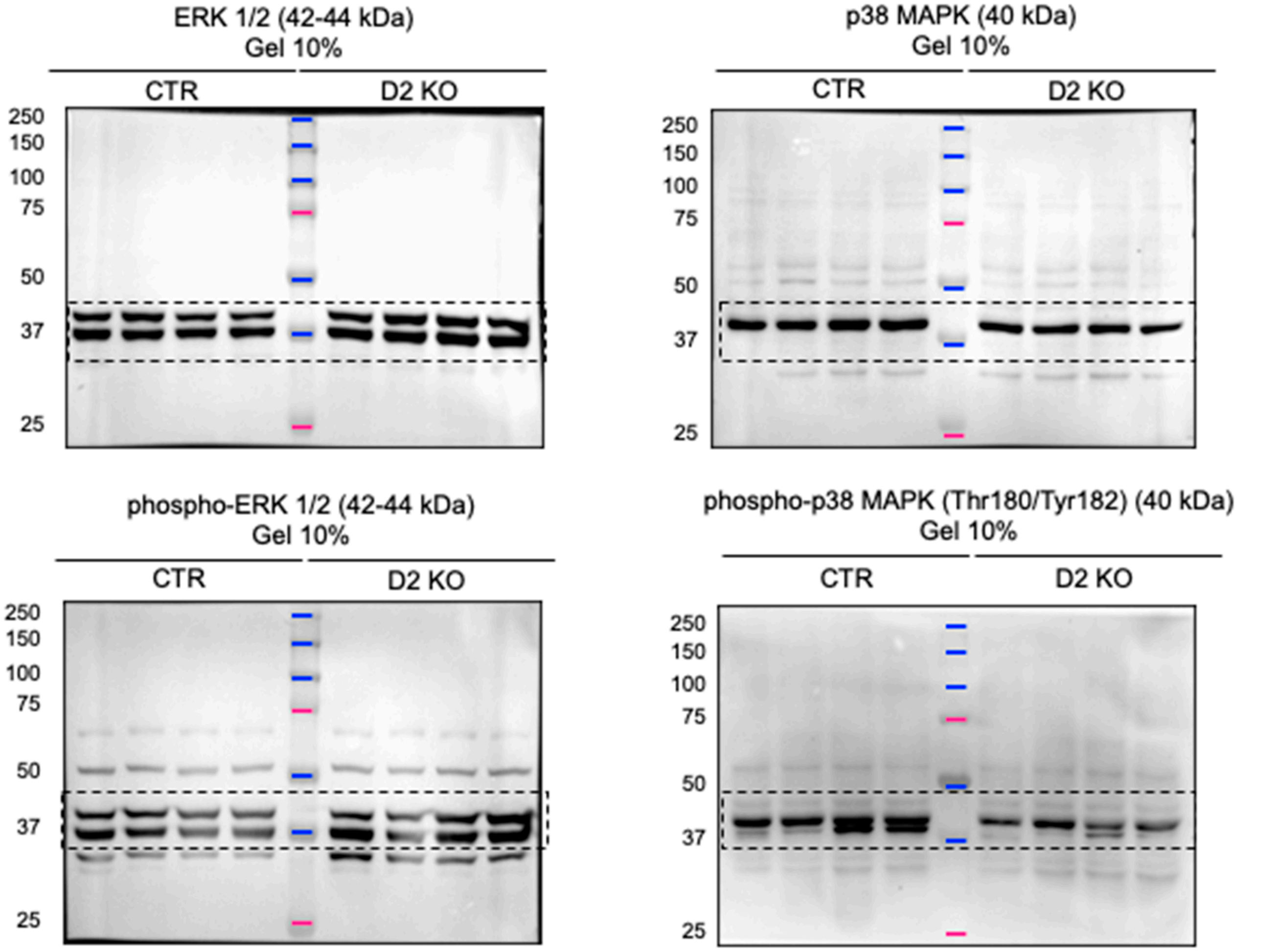

Precision Plus Protein Standards:
Dual Color BIORAD #161-0374

Figure S5B

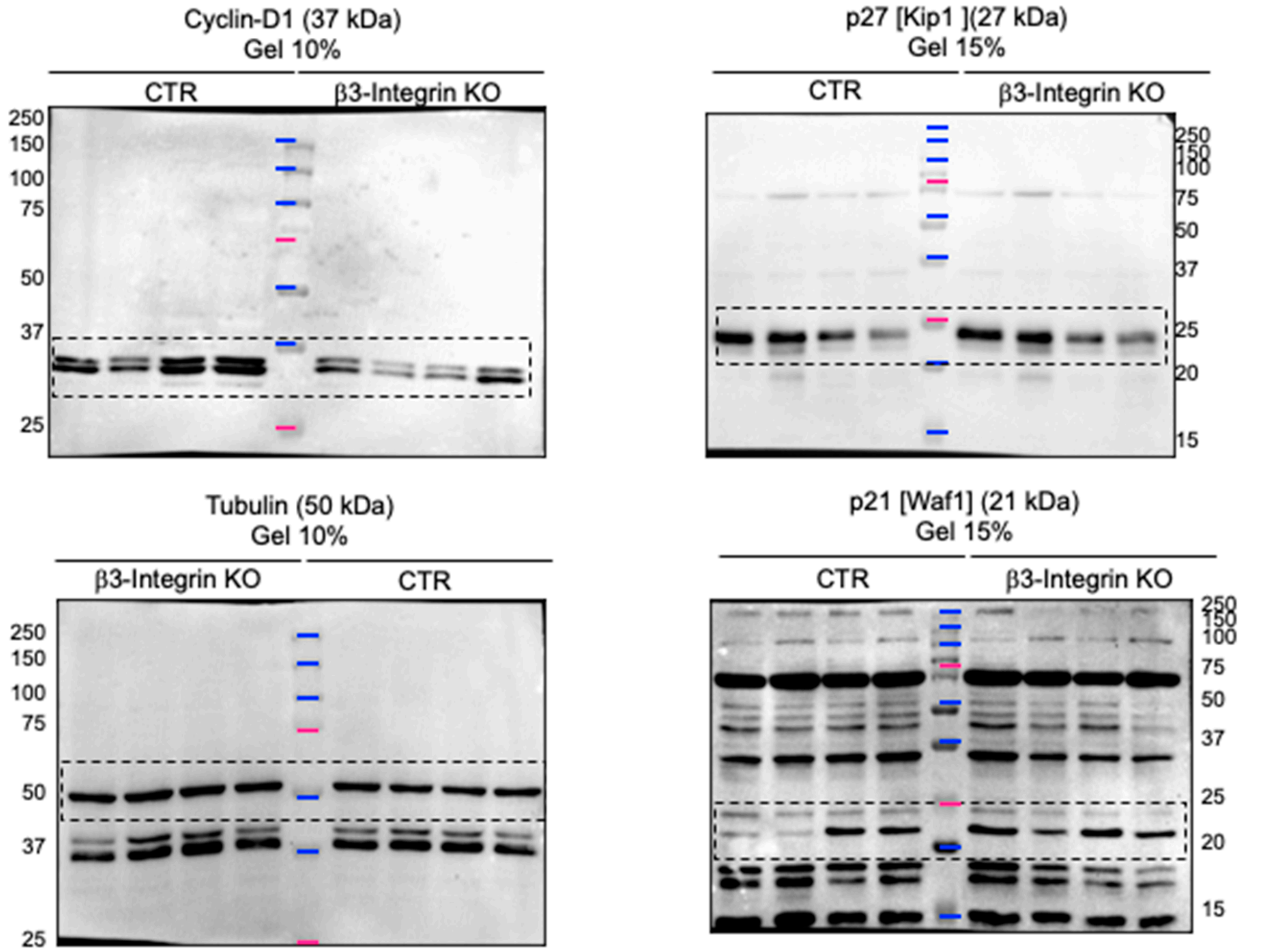

Precision Plus Protein Standards:
Dual Color BIORAD #161-0374
